# Aberrant Cholesterol Metabolic Genes Regulation in a Negative Feedback Loop Induced by an Alphacoronavirus

**DOI:** 10.3389/fnut.2022.870680

**Published:** 2022-03-18

**Authors:** Hao-Yu Liu, Haotian Gu, Huan Qu, Wenbin Bao, Yanhua Li, Demin Cai

**Affiliations:** ^1^College of Animal Science and Technology, Yangzhou University, Yangzhou, China; ^2^College of Veterinary Medicine, Yangzhou University, Yangzhou, China

**Keywords:** cholesterol, SREBP2, FXR, epigenetic, PEDV, coronaviruses

## Abstract

Porcine epidemic diarrhea virus (PEDV) is an alphacoronavirus that causes acute inflammation and severe diarrhea in newborn piglets with a high mortality rate. Given that cholesterol is required for coronavirus infection *in vitro*, the role of endogenous cholesterol metabolism in regulating coronavirus infection and the mechanism behind it ought to be elucidated. In this study, we found that the levels of cholesterol and bile acids were both elevated in the livers of PEDV-infected piglets compared to those of the control group. Consistently, in the livers of PEDV-infected piglets, the expression of key genes involved in cholesterol metabolism was significantly increased. Transcriptomic analysis indicated that the cholesterol homeostasis pathway was among the most enriched pathways in the livers of PEDV-infected piglets. Unexpectedly, the expression of key genes in the cholesterol metabolic pathway was downregulated at the messenger RNA (mRNA) level, but upregulated at the protein level. While the primary transcriptional factors (TFs) of cholesterol metabolism, including SREBP2 and FXR, were upregulated at both mRNA and protein levels in response to PEDV infection. Further Chromatin Immunoprecipitation Quantitative Real-time PCR (ChIP-qPCR) analysis demonstrated that the binding of these TFs to the locus of key genes in the cholesterol metabolic pathway was remarkably inhibited by PEDV infection. It was also observed that the occupancies of histone H3K27ac and H3K4me1, at the locus of the cholesterol metabolic genes *HMGCR* and *HMGCS1*, in the livers of PEDV-infected piglets, were suppressed. Together, the PEDV triggers an aberrant regulation of cholesterol metabolic genes *via* epigenetic inhibition of SREBP2/FXR-mediated transcription, which provides a novel antiviral target against PEDV and other coronaviruses.

## Introduction

Coronaviruses (CoVs) primarily infect birds and mammals; some of them lead to zoonotic diseases in humans, which has been a public health concern. During the past two decades, several emerging coronaviruses have caused severe diseases to humans and animals, such as Severe Acute Respiratory Syndrome Coronavirus (SARS-CoV) in 2002 (1), Middle East Respiratory Syndrome Coronavirus (MERS-CoV) in 2012 (2), the highly pathogenic PEDV variants in 2013 (3), and SARS-CoV-2 in 2019 (4). To date, the coronavirus disease 2019 (COVID-19) pandemic of SARS-CoV-2 infection has caused an unprecedented crisis in global healthcare systems (5). Notably, on rare occasions, some CoVs from animals could cross the animal-human species barrier and establish zoonotic diseases in humans. Porcine epidemic diarrhea virus (PEDV) can infect pigs at all ages and cause grievously contagious enteric diseases with a mortality rate of newborn piglets up to 100% (3). The PEDV is a single-stranded positive-sense RNA virus and an alphacoronavirus in the family *Coronaviridae*, order *Nidovirales* (6). This virus contains four major structural proteins: the spike (S) protein, envelope (E) protein, matrix (M) protein, and nucleocapsid (N) proteins (7, 8). The PEDV is usually transmitted by the fecal-oral route, or through the air from the nasal cavity to the intestinal mucosa (9, 10).

As the unique liquid-ordered microenvironments in the plasma membrane, lipid rafts play a critical role in cellular physiological homeostasis during the virus life cycle (11). Lipid rafts are accumulated with cholesterol, which facilitates the maintenance of the tight sphingolipids packaging. Cholesterol depletion by MβCD could cause structural disorder and disorganization of the lipid raft (12). Plasma membrane cholesterol is required for the infection processes of non-enveloped and enveloped viruses. Since CoVs have a lipid envelope, cholesterol biosynthesis (CB) exerts functions in the steps of viral attachment, replication, and assembly (11). Moreover, it is documented that cellular cholesterol is important for PEDV infection *in vitro* (12). Therefore, cholesterol metabolism is a critical pathway for antiviral therapeutics to CoVs. Indeed, cholesterol-lowering drugs like statins and fibrates have been reported to inhibit SARS-CoV-2 infection (13, 14). Cholesterol 25-hydroxylase which is an enzyme to catalyze the oxidized form of cholesterol to 25-hydroxycholesterol is proved to be a natural host restriction factor against PEDV infection (15). On the contrary, a recent study reveals that lower blood concentrations of total cholesterol are correlated with more severe disease and increased mortality in patients with COVID-19 (16). Thus, further investigations are required to fully understand the relationship between cholesterol metabolism and coronavirus infection.

The liver, as the central metabolic organ, is the main site of CB (17, 18). The CB is primarily modulated by the TFs' sterol regulatory element-binding protein 2 (SREBP2) and farnesoid X receptor (FXR) (19–21). When low cholesterol levels are detected in the endoplasmic reticulum, SREBP2 is cleaved, translocated into the cell nucleus, and bound to sterol response elements to activate the expression of enzymes associated with cholesterol biosyntheses, such as 3-hydroxy-3-methylglutaryl-CoA reductase (HMGCR), 3-hydroxy-3-methylglutaryl-CoA synthase (HMGCS), and 24-dehydrocholesterol reductase (DHCR24) (22–24). On the contrary, SREBP2 is inactivated when cholesterol level increases at the cell membrane. Furthermore, cholesterol transformation is critical for maintaining hepatic cholesterol homeostasis. Cholesterol-7a-hydroxylase (CYP7a1) and cholesterol-27a-hydroxylase (CYP27a1) are the main enzymes catalyzing this biotransformation (20). It is modulated by nuclear transcription factor FXR in a negative-feedback loop, similar to that of SREBP2 (21). In recent years, the development of epigenetics provides new insights to solve the mechanism of coronavirus infection (25). Given that CB is valuable for epigenetic modulation of gene transcription involving histones modification (24, 26, 27), it is important to understand whether and, if so, how epigenetic mechanisms control CB in PEDV infection and the genetic networks behind. In this study, we aimed to uncover the mechanism of cholesterol metabolism regulated by PEDV infection in piglets. We found that the binding of SREBP2 and FXR to the locus of key genes in the pathways of cholesterol biosynthesis and cholesterol transformation was suppressed by PEDV infection, which results in failing to activate the expression of those genes at the messenger RNA (mRNA) level. Furthermore, we explored the epigenetic mechanisms involved in the abnormal programming of cholesterol metabolic genes during this alphacoronavirus infection.

## Results

### Cholesterol Metabolism Is Modulated in Piglet Livers by PEDV Infection

**Figure 1:**
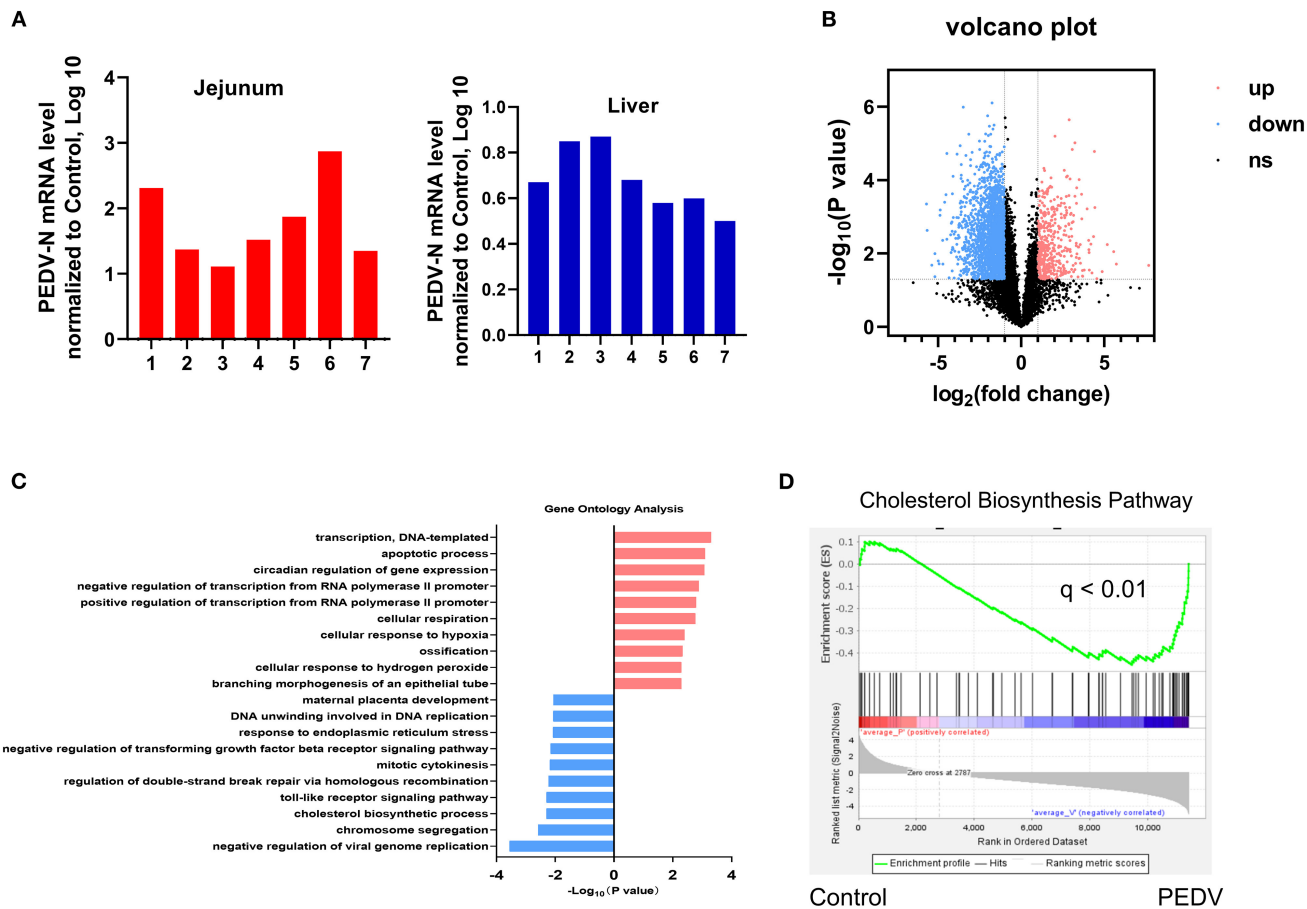
Cholesterol metabolism was modulated by Porcine epidemic diarrhea virus (PEDV) infection indicated by transcriptional profiling of piglet livers. **(A)** PEDV RNA expression was determined by Quantitative Real-Time PCR (qRT-PCR) analysis in the jejunum and liver tissues of PEDV-infected and control piglets. **(B)** Volcano plot visualization of the hepatic transcriptome measured by RNA-seq analysis. **(C)** Genes involved in the cholesterol biosynthesis pathway were among the most enriched pathways based on gene ontology (GO) analysis. **(D)** The gene-set enrichment analysis (GSEA) plot of the differentially expressed genes in the cholesterol biosynthesis pathway of livers from PEDV-infected piglets compared to the controls. False-discovery rate (FDR).

A quantitative reverse transcription PCR (qRT-PCR) assay targeting the PEDV N gene was conducted to detect viral RNA in the jejunum and liver samples collected from both healthy and PEDV-infected piglets. As expected, viral RNA was detected in the jejunum and liver samples of PEDV-infected piglets, but not in those of control piglets (Figure 1A). To identify the effect of PEDV infection on the core transcription programs, RNA-seq analysis was performed using the liver tissues of PEDV-infected piglets and control piglets, respectively. The differentially expressed genes in the hepatic transcriptome between the two groups were visualized by a volcano plot. We found that the number of downregulated genes was significantly larger than that of the upregulated genes in the livers of PEDV-infected piglets (Figure 1B). the Gene Ontology (GO) analysis of the most downregulated 1,500 transcripts in the livers of PEDV-infected piglets showed that the cholesterol biosynthesis pathway was among the most enriched pathways (Figure 1C). The gene-set enrichment analysis (GSEA) also indicated that the hallmarks of the cholesterol-homeostasis pathway were strongly altered by PEDV infection (Figure 1D).

### The Expression of Genes in the Cholesterol Biosynthesis Pathway vs. the Cholesterol Content in the PEDV-Infected Piglets

**Figure 2:**
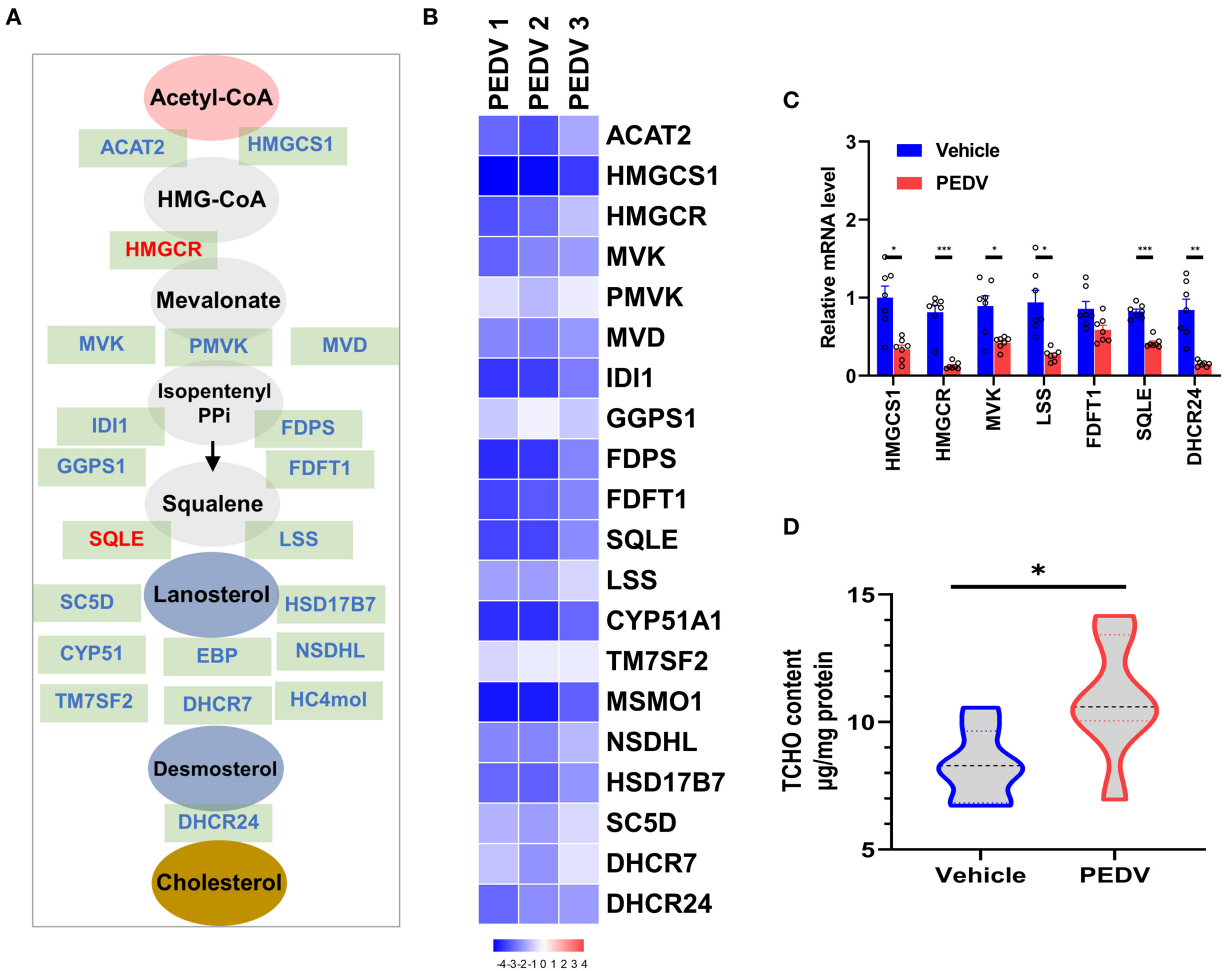
The expression of genes in the cholesterol biosynthesis pathway and cholesterol content in piglet livers. **(A)** The graphic scheme of the cholesterol biosynthesis pathway from acetyl-CoA. The core genes involved in cholesterol biosynthesis were highlighted in blue, while two rate-limiting enzymes 3-hydroxy-3-methylglutaryl-CoA reductase (HMGCR) and SQLE were highlighted in red. **(B)** Heatmap of messenger RNA (mRNA) expression (RNA-seq) changes of the cholesterol biosynthesis genes in the livers of PEDV-infected piglets (log2 transformed, normalized to Control). **(C)** The qRT-PCR analysis confirmed changes of the cholesterol biosynthesis genes in the livers of PEDV-infected piglets. **(D)** Total cellular cholesterol contents in the livers of the control and PEDV-infected piglets were analyzed and normalized to total protein concentrations. The data are shown as the means ± SEM, *P < 0.05, ** *P* < 0.01, ***P < 0.001, using the two-tailed Student *t*-test.

**Figure 3:**
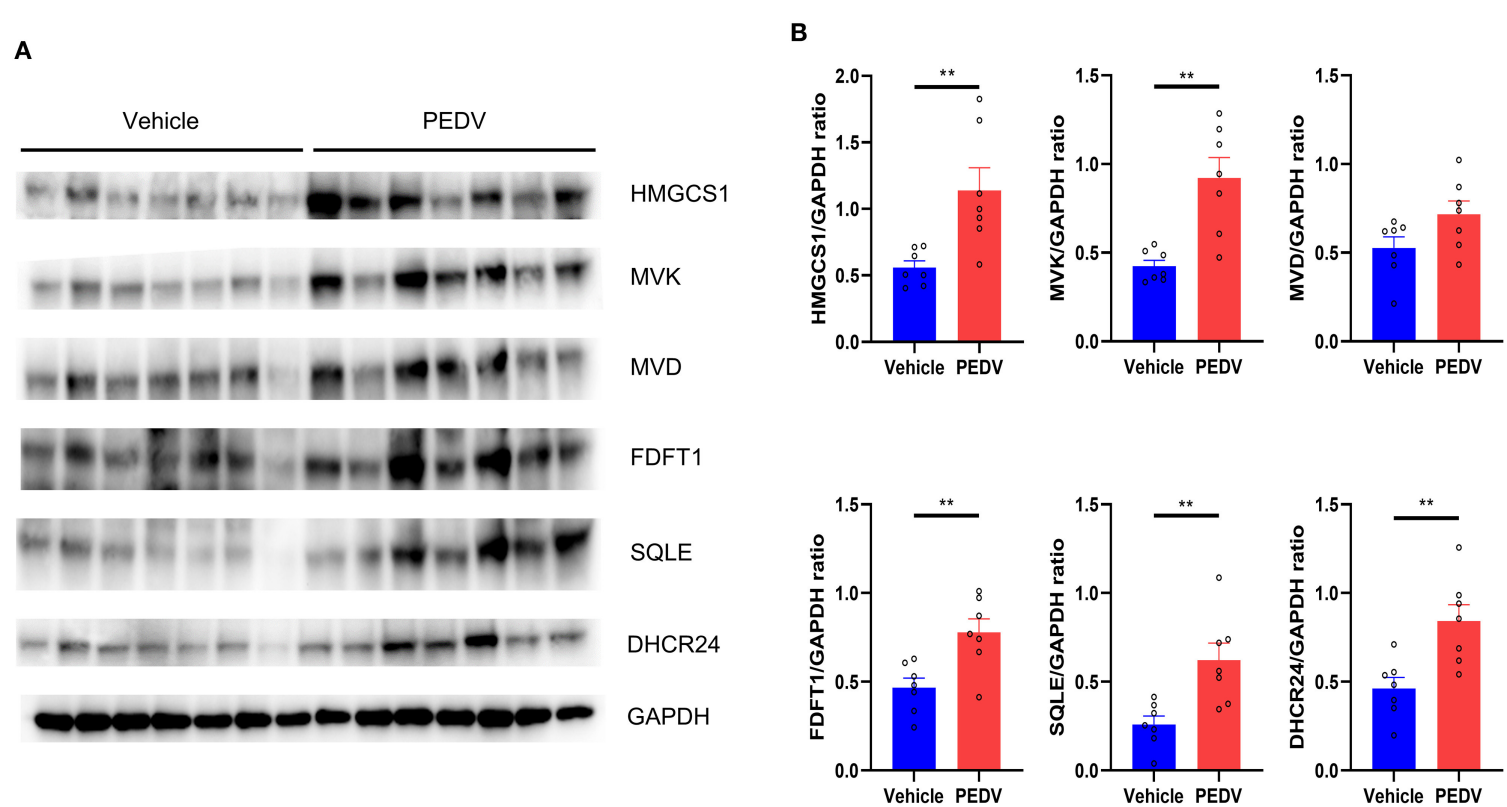
The expression of core genes in the cholesterol biosynthesis pathway was upregulated at the protein level. **(A)** Western blotting analysis was conducted to evaluate the expression of HMGCS, MVK, MVD, FDFT1, SQLE, and 24-dehydrocholesterol reductase (DHCR24). **(B)** The relative expression levels of these genes were normalized to the housekeeping gene GAPDH. The data are shown as the means ± SEM, *n* = 7, **P < 0.01, using the two-tailed Student *t*-test.

It is well-known that at least 21 enzymes are involved in the cholesterol-biosynthesis program to generate cholesterol (Figure 2A). Among them, the HMGCR and Squalene Epoxidase (SQLE) are the rate-limiting enzymes to determine the cholesterol synthesis rate and cholesterol level in circulation. The pathway-focused data analysis exhibited that a vast majority of the cholesterol-synthesis genes were strongly downregulated in the PEDV-infected piglets (Figure 2B). The transcriptional inhibition of key genes *3-Hydroxy-3-Methylglutaryl-CoA Synthase 1 (HMGCS1), HMGCR, Mevalonate Kinase (MVK), Lanosterol Synthase (LSS), Farnesyl-Diphosphate Farnesyltransferase 1 (FDFT1), SQLE*, and *DHCR24* was further validated by the qRT-PCR analysis (Figure 2C). Intriguingly, in contrast to the reduced expression of genes in the cholesterol biosynthesis pathway, we found that PEDV infection significantly increased the cholesterol content in the livers (Figure 2D). To figure out the incoordination between cholesterol content and the mRNA expression of cholesterol biosynthesis genes, we determined the expression of core enzymes in the CB pathway at the protein level. In agreement with the elevated cholesterol content, the PEDV infection strongly upregulated the expression of the core enzymes at the protein level, including HMGCS1, MVK, Mevalonate Diphosphate Decarboxylase (MVD), FDFT1, SQLE, and DHCR24 (Figures 3A,B).

### PEDV Infection Modulates the Pathway of Cholesterol Transformation to Bile Acids

**Figure 4:**
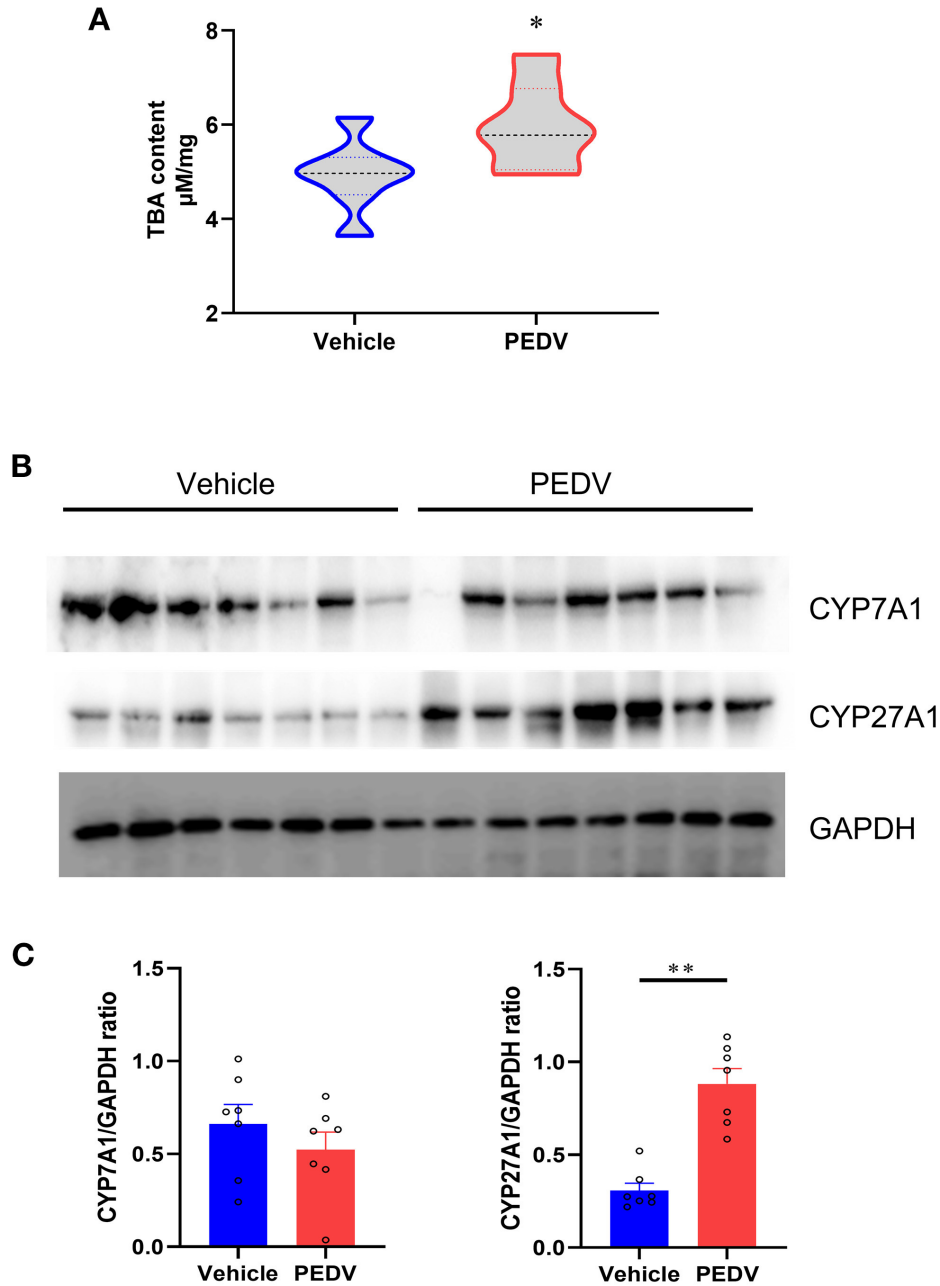
Total bile acids (TBA) content and the expression of genes involved in cholesterol transformation were elevated in livers of PEDV-infected piglets. **(A)** TBA contents in the livers of piglets were analyzed and normalized to total protein concentrations. **(B,C)** Western blotting analysis was performed to evaluate the expression of Cholesterol-7a-hydroxylase (CYP7A1) and cholesterol-27a-hydroxylase (CYP27A1) at the protein level. Based on the intensity of protein bands, the relative expression levels of CYP7A1 and CYP27A1 were determined by normalization to GAPDH. The data are shown as the means ± SEM, *n* = 7, *P < 0.05, **P < 0.01, using the two-tailed Student *t*-test.

**Figure 5:**
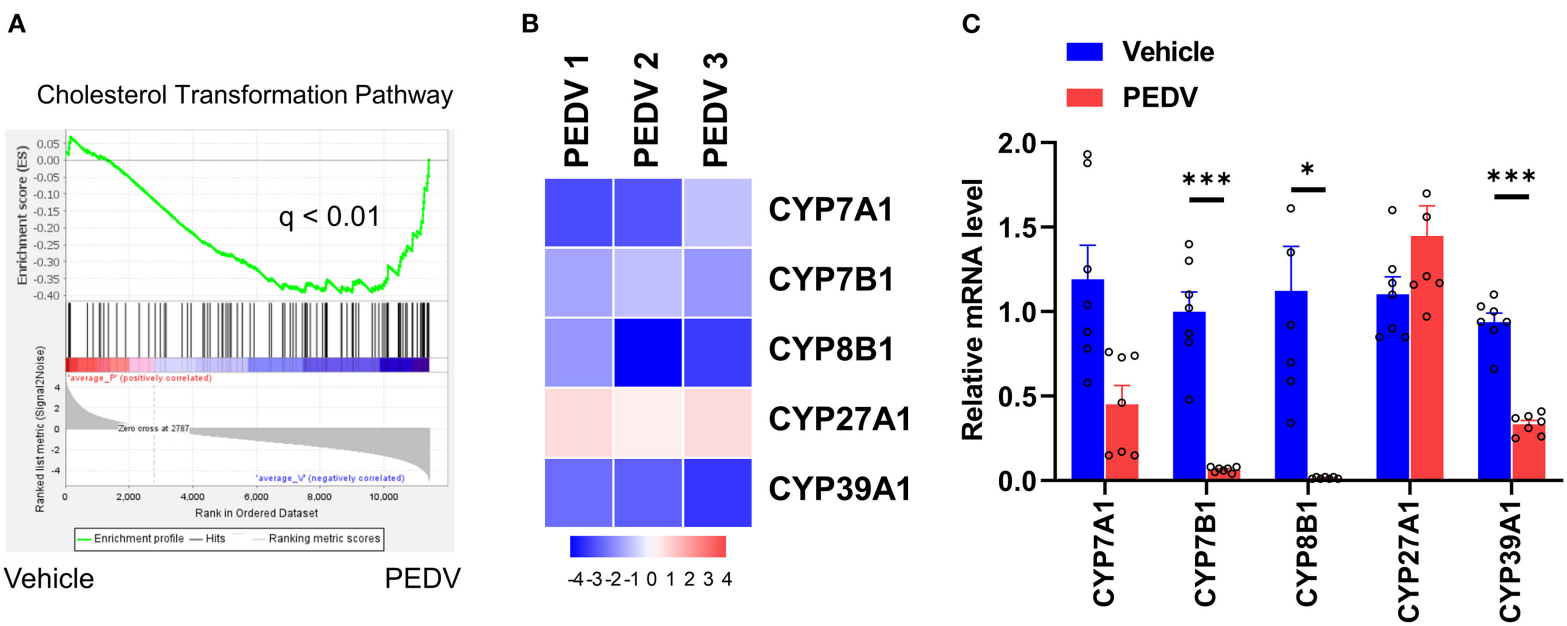
The expression pattern of genes involved in the cholesterol transformation pathway. **(A)** GSEA plots depicting the enrichment of genes in the cholesterol transformation pathway of livers from PEDV-infected piglets compared to the controls. FDR, false-discovery rate. **(B)** Heatmap of mRNA expression (RNA-seq) changes of the genes involved in cholesterol transformation to bile acids in the livers of PEDV-infected piglets (log2 transformed, normalized to Control). **(C)** The qRT-PCR analysis confirmed expression changes of genes in the cholesterol transformation pathway in the livers of PEDV-infected piglets. The data are shown as the means ± SEM, *P < 0.05, ***P < 0.001, using the two-tailed Student *t*-test.

Bile acids are the end products of cholesterol catabolism. Herein, we examined the total bile acid (BA) content in the livers of piglets. Consistent with the increased total cholesterol content, the total BA content in the liver was raised in the piglets infected with PEDV (Figure 4A). We further determined the expression of the rate-limiting enzymes CYP7A1 and CYP27A1 at the protein level, which represent the classical and alternative pathways of bile acid biosynthesis respectively. In line with liver BA contents, we observed that the alternative pathway was remarkably activated because of the highly CYP27A1 expression at the translational level (Figures 4B,C). However, the CYP7A1 protein content was not changed in response to PEDV infection. Similar to genes in the cholesterol biosynthesis pathway, the genes involved in the BAs-biosynthesis pathway was among the most enriched program and was downregulated in the PEDV-infected piglets using GO and GSEA analysis (Figure 5A). The pathway-focused results revealed that the key genes functional in cholesterol transformation were strongly downregulated in the PEDV-infected piglets (Figure 5B). The transcriptional inhibition of these genes was also validated by the qRT-PCR analysis (Figure 5C).

### PEDV Infection Epigenetically Inhibits SREBP2 and FXR Transcriptional Activation

**Figure 6:**
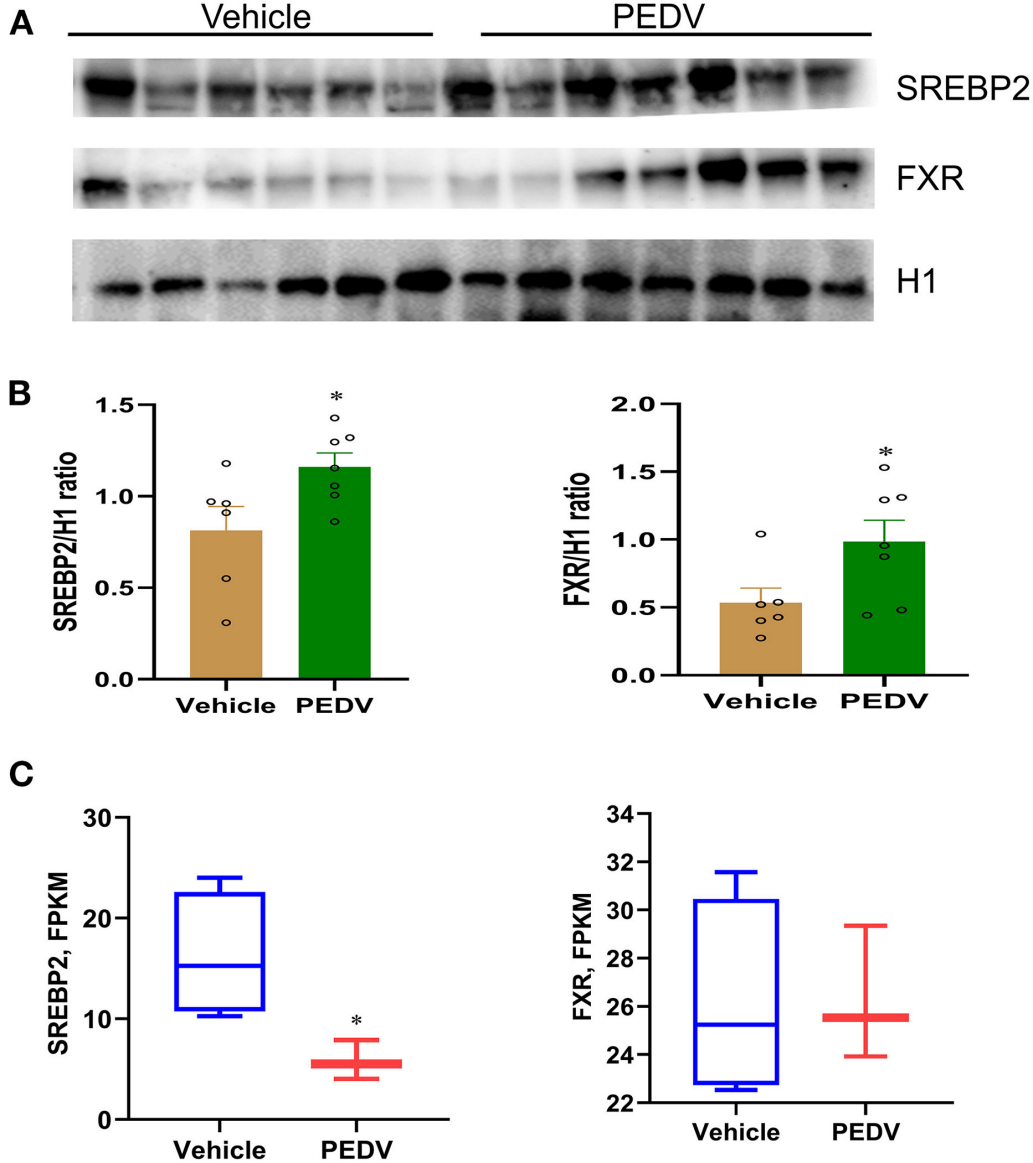
The nuclear expression of SREBP2 and FXR was upregulated in the livers of PEDV-infected piglets. **(A,B)** Western blotting analysis was performed to evaluate the expression of nuclear SREBP2 and FXR at the protein level. Based on the intensity of protein bands, the expression of SREBP2 and FXR were normalized to that of histone H1, *n* = 6–7. **(C)** Graphic summary of mRNA expression of sterol regulatory element-binding protein 2 (SREBP2) and farnesoid X receptor (FXR) by RNA-seq analysis, *n* = 3–4. The data are shown as the means ± SEM, *P < 0.05, using the two-tailed Student *t*-test.

**Figure 7:**
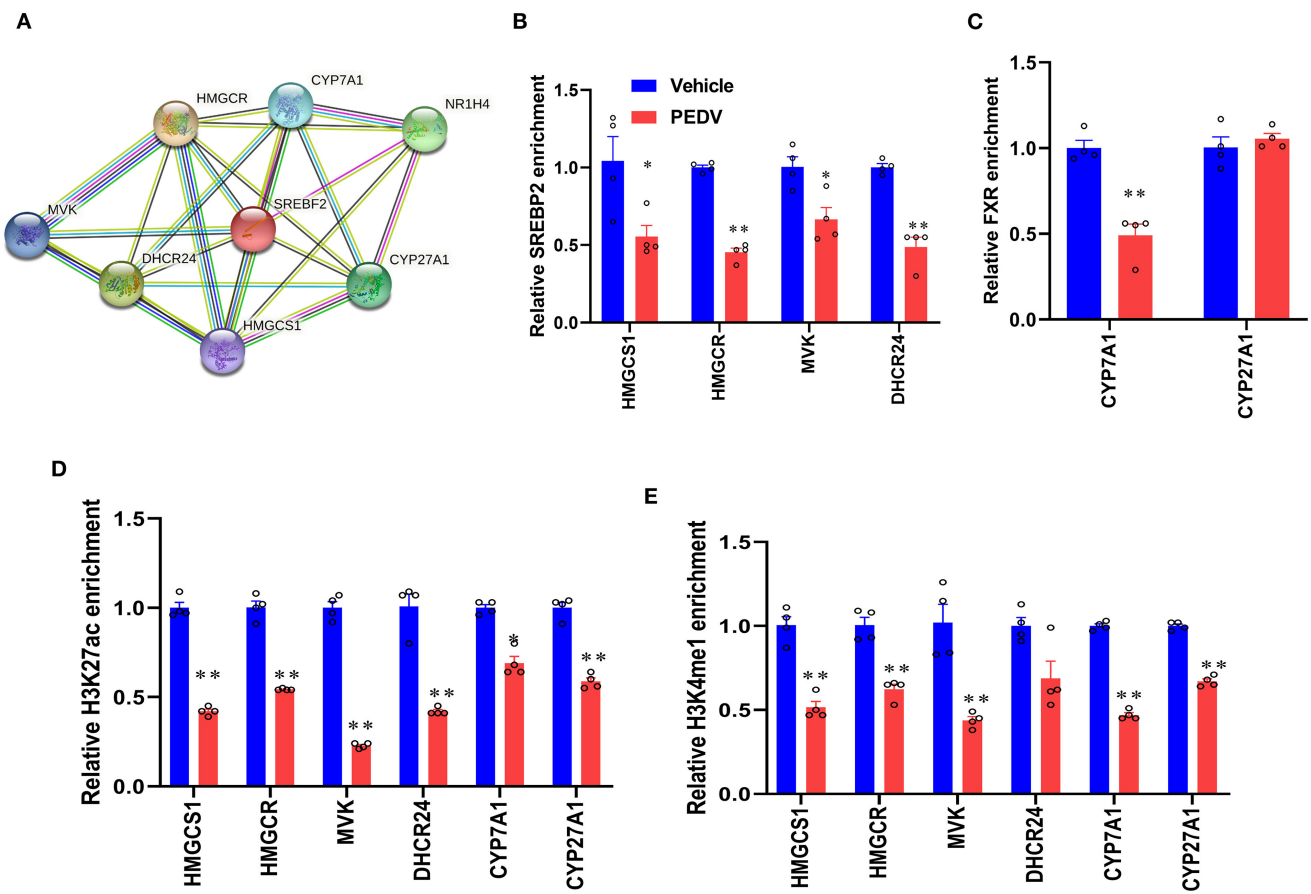
PEDV infection epigenetically inhibited the transcriptional activation of gene expression by SREBP2 and FXR. **(A)** The interactions among SREBP2, FXR, and core proteins involved in cholesterol metabolism during transcriptional regulation were predicted by Search Tool for the Retrieval of Interacting Genes (STRING). **(B,C)** The relative enrichment of SREBP2 and FXR at the locus of indicated genes in livers was analyzed by ChIP-qPCR. **(D,E)** The relative enrichment of histone H3K27ac and H3K4me1 occupancy was analyzed by ChIP-qPCR. The data are shown as the means ± SEM, *P < 0.05, ** *P* < 0.01 using the two-tailed Student *t*-test.

To maintain cholesterol homeostasis, both cholesterol and bile acids modulate the biosynthesis pathway in a negative-feedback manner by SREBP2 and FXR, respectively. In this regard, we hypothesize that the higher concentrations of cholesterol and bile acids would inhibit the nuclear translocation of these two factors. However, the results of western blot analysis showed that their expression was dramatically elevated in the cell nucleus of the PEDV-infected piglets (Figures 6A,B). Therefore, the markedly downregulated gene transcripts would be attributed to the loss of TFs' binding occupancies. Interestingly, the mRNA level of SREBP2 was decreased, while that of FXR was not changed in the PEDV-infected piglets (Figure 6C). The potential interactions among SREBP2, FXR, and the key enzymes involved in cholesterol metabolism during transcriptional regulation were predicted by Search Tool for the Retrieval of Interacting Genes (STRING) analysis from the European life-sciences Infrastructure for biological Information (ELIXIR) database (Figure 7A). To uncover the mechanism underlying the function of SREBP2 and FXR, we performed a ChIP-qPCR analysis of these two TFs in the livers of the two groups. Indeed, a strong reduction of SREBP2 and FXR binding was observed at promoters of their major targets, including *HMGCS1, HMGCR, MVK, DHCR24*, and *CYP7A1* (Figures 7B,C). In concomitant with the loss of TFs' enrichments, the transcriptional activation-linked histone mark Histone H381(acetyl K27) (H3K27ac) and Histone H3 (mono methyl K4) (H3K4me1) were also diminished (Figures 7D,E). Collectively, these results implied that in the PEDV-infected piglets, histones modification exerts a pivotal role in inhibiting the expression of genes involved in cholesterol-biosynthesis and transformation *via* blunting the specific chromatin-DNA binding by two key TFs' SREBP2 and FXR.

## Discussion

To date, the coronaviruses have brought numerous illnesses, including enteritis in livestock, upper respiratory diseases in birds, and potentially lethal respiratory infections in humans (28), the epidemiology of PEDV is still extremely significant, with morbidity and mortality rates of piglets up to 100% (29). Despite the classic clinical symptoms of PEDV infection, such as watery diarrhea and vomiting, increasing research now pays more attention to the host physiology and homeostasis, like growth performance alteration, tissue accretion, and organ damages caused by virus infection (30, 31). In this regard, studies on porcine alphacoronaviruses are necessary not only for exploring strategies to control their infection in pig populations but also for understanding the underlying molecular mechanisms. Here, we found that cholesterol and its transformed products were both increased in the livers of the PEDV-infected piglets. This should be attributed to the elevated key enzymes involved in cholesterol metabolism, including HMGCS1, MVK, MVD, FDFT1, SQLE, DHCR24, and CYP27A1. Two main transcription factors, the SREBP2 and FXR, were highly upregulated at the translation level in the cell nucleus. Given the importance of the cholesterol balance, the overt cholesterol contents negatively inhibit the transcripts of the CB and cholesterol transformation pathways reflected by the transcriptomic results. However, this negative-feedback loop did not regulate through the well-known inhibition of the nuclear translocation of SREBP2 and FXR, but *via* the loss of DNA-binding at the transcription stage. We also demonstrated that histone repressive modifications *via* diminishing H3K27ac and H3K4me1 facilitate the trans-inactivation of SREBP2 and FXR.

It is well-known that cholesterol is an essential lipid component of cell membranes, thus, cholesterol depletion blocks the virus entry possibly due to lipid rafts (12). Lipid rafts are sub-domains of the plasma membrane enriched with cholesterol and glycosphingolipids. Often, lipid rafts play a couple of roles during coronavirus infection; for instance, providing the platforms for specific interaction between coronavirus S protein and Angiotensin Converting Enzyme 2 (ACE2) receptor, and facilitating viral endocytosis (12, 32, 33). It is worth noting that the potential functions of cholesterol in the viral entry have been documented for numerous coronaviruses, including SARS-CoV (32), infectious bronchitis (34), murine coronavirus (35), and porcine delta coronavirus (12). Similar to the alphacoronavirus we observed, it is reported that cholesterol accumulation results in virus replication by promoting viral entry. Therefore, it is suggested that high cholesterol content is a critical indicator of coronaviruses infection (11). Alternatively, it is noted that cholesterol exerts a role in virus entry into the host cell when it binds and alters the oligomeric pattern of the N-terminal fusion peptide of the coronavirus S protein (36, 37). The viral mRNA levels were strongly reduced by cholesterol depletion on coronavirus infection (37). Interestingly, ion channels on viral particles are suggested to be involved in cholesterol-driven coronavirus infection because that cholesterol level is crucial for the lipid environment which benefits the charge in cellular membranes (38). Further investigations need to address the interactions between cholesterol and iron metabolism in PEDV infection.

As the central site for cholesterol metabolism, the liver is vital for the equilibrium of cholesterol among organs (26, 39). Although the intestine tissues are the primary sites where PEDV initiates infection, we also detected the mRNA in the liver of PEDV-infected piglets previously (40). Indeed, Wu et al. have revealed that the porcine liver is susceptible to PEDV, and liver damages were reflected by the significantly increased aspartate aminotransferase (AST), alanine aminotransferase (ALT), and AST/ALT ratio (30). Moreover, the PEDV induced the elevated expression of the key proteins involved in liver diseases (41). Cholesterol biosynthesis and transformation are the critical biochemical reactions to maintain metabolic circulation between the liver and the gut (42, 43). We observed that the key enzymes involved in these two pathways are upregulated in the PEDV-infected piglets, while enough cholesterol benefits the entry of PEDV into the hepatic cells. Given the fact that gluconeogenesis is augmented to produce more energy and nutrients to protect the liver and jejunum from virus infection (31), we reasonably thought that the activated CB is attributed to the increasing number of fuels in response to coronavirus invasion. In addition, in agreement with previous findings of coronavirus-triggered hyper-expression (44, 45), the upregulation of SREBP2 and FXR could initiate the activation of the CB pathway in PEDV-infected piglets. Another interesting finding of this study should be the incongruity between the mRNA and protein levels for CYP7A1 and CYP27A1 genes. The CYP7A1was downregulated at the mRNA level but increased at the protein level, while CYP27A1 was increased at the protein level but remained unchanged at the mRNA level. The dissociation of mRNA abundance and protein content implies the possible involvement of post-transcriptional mechanisms. Indeed, our and other previous studies have revealed that CYP7A1 and CYP27A1 are vulnerable to microRNAs-mediated post-transcriptional regulation (20, 46). However, it is still a question whether the dis-association we observed here is attributed to post-transcriptional modification during virus entry, which remains to be clarified in the future.

Despite that cholesterol lowering is a novel potential coronavirus therapeutic strategy, small-molecule inhibitors have previously been studied in the treatment of a variety of respiratory viral infections (47, 48). There is almost no evidence of a direct connection between statins and coronavirus infection. Moreover, several studies have shown that a downtrend in total cholesterol is likely a negative acute phase reactant response (16, 49). We must address that the doses of statin used for the treatment of hypercholesterolemia are generally safe, as we and others claim that cholesterol and bile acids are the special ligands to modulate CB homeostasis. The dramatic inhibition of the cholesterol and cholic biosynthesis genes is a clear manifestation of a strong blockade of the SREBP2/FXR-mediated negative feedback (21). The disconnection between cholesterol metabolic genes expression and SREBP2 has previously been observed in the COVID-19-infected model (44). In the present study, we did not see that PEDV inhibited the translation of SREBP2 protein into the nucleus. However, we mechanistically pointed out that the histone modifications may be the dominant event to suppress the generation of the transcriptional complex, which further reduced the transactivation of genes associated with cholesterol metabolic homeostasis.

In conclusion, the PEDV triggers aberrant regulation of cholesterol metabolic genes *via* epigenetic inhibition of SREBP2/FXR-mediated transcription, which provides a novel antiviral target against PEDV and other coronaviruses.

## Materials and Methods

### Animal Study Design

All experiments involving animals were reviewed and approved by the Institutional Animal Care and Use Committee of Jiangsu Province. Seven Large White piglets, naturally infected with PEDV featured with watery diarrhea and acute vomiting, together with seven control piglets as a negative control, were selected. All animals were raised under the same conditions and humanely euthanized for tissue collection. Liver tissues and jejunum tissues were sampled, snap-frozen in liquid nitrogen, and thereafter stored at −80°C until analysis.

### Measurement of Total Cholesterol (TCHO) and Total Bile Acids (TBA)

Liver tissues were washed three times with cold phosphate-buffered saline (PBS) and subjected to extraction with organic solvents (7:11:0.1, chloroform/isopropanol/Triton X-100). The TCHO and TBA were measured using the Tissue Total Cholesterol Assay Kit (E1015, Applygen, Beijing, China) and the total Bile Acid Assay Kit (STA-631, Cell Biolabs, Inc., CA, USA), and normalized to total protein concentrations.

### RNA-Seq Analysis

The RNA-seq libraries were generated as previously described (17), with modifications. The liver tissues of PEDV-infected piglets (*n* = 3) and control (*n* = 4) piglets were randomly selected and washed with cold PBS and subjected to total cellular RNA extraction. Total RNA (2 μg) was prepared using the Illumina Tru-Seq RNA Sample Prep Kit according to the manufacturer's instructions. The quality of libraries was checked with an Agilent Bioanalyzer (Agilent Technologies, Palo Alto, CA, USA). The high through sequencing was performed on an Illumina HiSeq 2000 sequencer at BGI Tech (Wuhan, China). The sequence data in FASTQ format was analyzed using standard BWA–Bowtie–Cufflinks workflow as described previously (24). Briefly, sequence reads were mapped to susScr3 assembly with BWA and Bowtie software. The Cufflinks package was used for transcripts assembly, quantification of normalized gene and isoform expression, and analysis of differentially expressed genes. The Gene Set Enrichment Analysis (GSEA v.3.0) was applied to rank genes based on the shrunken limma log2 fold changes. The GSEA tool was used in the “pre-ranked” model with default parameters. A gene ontology analysis was performed using DAVID Bioinformatics Resources 6.8.

### qRT-PCR Analysis

The total RNA extracted from liver tissues of control and PEDV-infected piglets using the TRIzol Reagent (Invitrogen, MA, USA) was reverse-transcribed to single-stranded DNA (cDNA) using the HiScript II Q RT SuperMix (Vazyme biotech, Nanjing, China) according to the manufacturer's instructions. The purity and concentration of total RNA were evaluated by electrophoresis in 1% agarose gel and NanoReady Spectrophotometer (Suizhen, Hangzhou, China). The qRT-PCR analysis was carried out on an ABI QuantStudio 3 Real-Time PCR Instrument (Applied Biosystems) using the SYBR Green Master Mix (Vazyme Biotech, Nanjing, China). The 10 μl reaction mixture contains 5 μl of AceQ qPCR SYBR Green Master Mix (2 ×),0.2 μl of ROX Reference Dye II (50 ×), 1 μl of cDNA template,0.2 μl of forward primer (10 μmol/L), and reverse primer (10 μmol/L), and 3.4 μl of ddH_2_O. The Glyceraldehyde-3-Phosphate Dehydrogenase (GAPDH) mRNA was detected as an internal reference to normalize the expression level of each transcript. The relative expression levels of indicated genes were calculated using the ΔΔCt method.

### Western Blotting Analysis

Liver tissues of PEDV-infected and control piglets were lysed with 500 μl cell lysis buffer for western blotting (Biosharp, Hefei, China) supplemented with phosphatase and protease inhibitor (Beyotime, Nanjing, China) according to the manufacturer's instructions. The tissue debris was removed by centrifugation at 12,000 rpm for 10 min at 4°C, cellular proteins in the supernatant were collected and separated in 10% sodium dodecyl sulfate polyacrylamide gel electropheresis (SDS-PAGE) gel. Next, the separated proteins were transferred to Polyvinylidene Fluoride (PVDF) membranes (Millipore, CA, USA). After being blocked with 5% skimming milk, the membranes were incubated with primary antibodies overnight at 4°C, respectively. The membranes were further incubated with an Horseradish Peroxidase (HRP)-conjugated secondary antibody. Finally, the membranes were developed with a Chemiluminescent Western Blot Detection kit (Vazyme Biotech, Nanjing, China) using Tanon 5200 Multi imaging system.

The nuclear protein was extracted from a 200 mg frozen liver sample as described previously, with modifications [54]. Briefly, the tissues were washed three times and then lysed with lysis buffer (10 mM N-2-hydroxyethylpiperazine-N-ethanesulphonicacid (HEPES), pH 7.9, 10 mM KCl, 0.1 mM Horseradish Peroxidase (EDTA), 0.4% NP-40, and protease inhibitor cocktail) for 30 min at 4°C. The homogenates were centrifuged for 30 s at 15,000 × g at 4°C. The supernatant was removed. The pellets were lysed in extraction buffer (20 mM HEPES, pH 7.9, 0.4 M NaCl, 1 mM EDTA, and protease inhibitor cocktail) for 15 min for nuclear extract collection.

### ChIP-QPCR

The ChIP-qPCR was performed as previously described (26, 27), with modifications. Briefly, the liver tissues of PEDV-infected piglets and control piglets were ground into powders with a mortar, then, resuspended in fixing buffer (50 mmol/L HEPES-KOH (Potassium Hydroxide), 100 mmol/L NaCl, 1 mmol/L EDTA, and 0.5 mol/L Ethylene Glycol Tetraacetic Acid (EGTA)) before being subjected to cross-linking with 1% formaldehyde for 5 min, followed by quenching with glycine on ice for 6 min. The precipitation was collected by centrifugation and resuspended in lysis buffer (50 mmol/L HEPES pH 8, 140 mmol/L NaCl, 1 mmol/L EDTA, 10% glycerol 0.5% NP40, 0.25% Triton X-100). The precipitation was then resuspended in washing buffer (10 mmol/L Tris pH 8, 1 mmol/L EDTA, 0.5 mmol/L EGTA, and 200 mmol/ L NaCl), washed, and resuspended in shearing buffer (0.1% SDS, 1 mmol/L EDTA, pH 8, 10 mmol/L Tris HCl, and pH 8) before sonication using Covaris M220 following the manufacturer's instructions. After being precipitated using gene-specific antibodies and protein G-conjugated beads, the chromatin fragments were treated with RNase A and proteinase K. Then, a purified ChIP DNA was used for qPCR analysis.

### Statistical Analysis

Statistical analyses were performed with the GraphPad Prism software 8.0. The data are presented as mean values ± SEM from at least three independent experiments. Statistical analysis was performed using two-tailed Student's *t*-tests or ANOVA with Tukey's *post hoc* test to compare the means. The value of *P* < 0.05 was considered significant.

## Data Availability Statement

The datasets presented in this study can be found in online repositories. The names of the repository/repositories and accession number(s) can be found in the article/supplementary material.

## Ethics Statement

The animal study was reviewed and approved by the Animal Care and Use Committee of Yangzhou University (YZUDWSY 2017-09-06).

## Author Contributions

DC and YL conceptualized the study. H-YL, HG, and HQ contributed to methodology and investigation. H-YL and DC wrote the original draft. H-YL, YL, WB, and DC reviewed and edited the manuscript. DC contributed to funding acquisition and supervision. H-YL, HG, and DC contributed to resources. All authors contributed to the article and approved the submitted version.

## Funding

This work was supported by the National Natural Science Foundation of China (32002243), Natural Science Foundation of Jiangsu Province (BK20200932), Natural Science Foundation of the Higher Education Institutions of Jiangsu Province (20KJB230001), the Jiangsu Agricultural Science And Technology Innovation Fund [CX(21)2014 and CX(21)3125], and the Priority Academic Program Development of Jiangsu Higher Education Institutions (PAPD).

## Conflict of Interest

The authors declare that the research was conducted in the absence of any commercial or financial relationships that could be construed as a potential conflict of interest.

## Publisher's Note

All claims expressed in this article are solely those of the authors and do not necessarily represent those of their affiliated organizations, or those of the publisher, the editors and the reviewers. Any product that may be evaluated in this article, or claim that may be made by its manufacturer, is not guaranteed or endorsed by the publisher.
